# Supplementary material for: Variability in Provider Assessment of Sepsis and Potential of Host Response Technology to Address this Dilemma—Results of an Online Delphi Study
Source: J Pers Med. 2023 Dec 5;13(12):1685. doi: 10.3390/jpm13121685 (PMC10744443; doi:10.3390/jpm13121685)
Supplement: Supplementary file 1 [file jpm-13-01685-s001.zip › jpm-2658080-supplementary.pdf]

Supplemental Materials

Supplemental Figure S1. Trends across specialties in perceived pre-test probabilities for Patient A

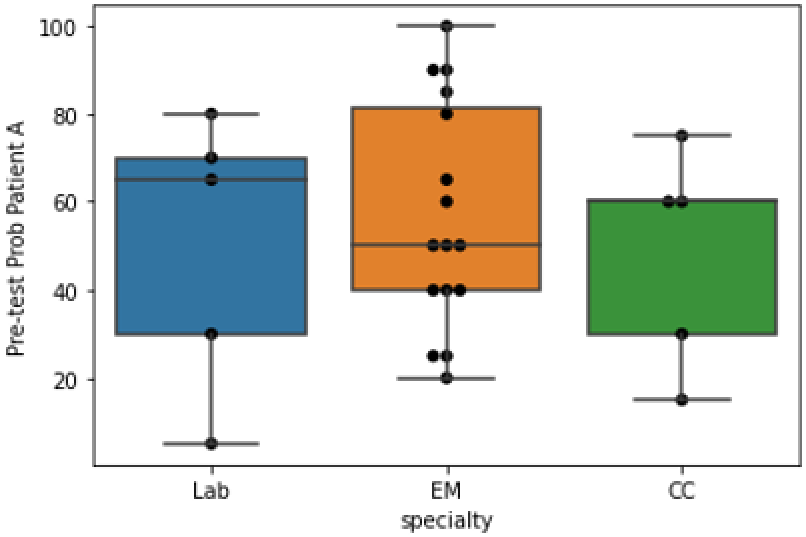

Supplemental Figure S2. Trends across specialties in perceived pre-test probabilities for Patient B

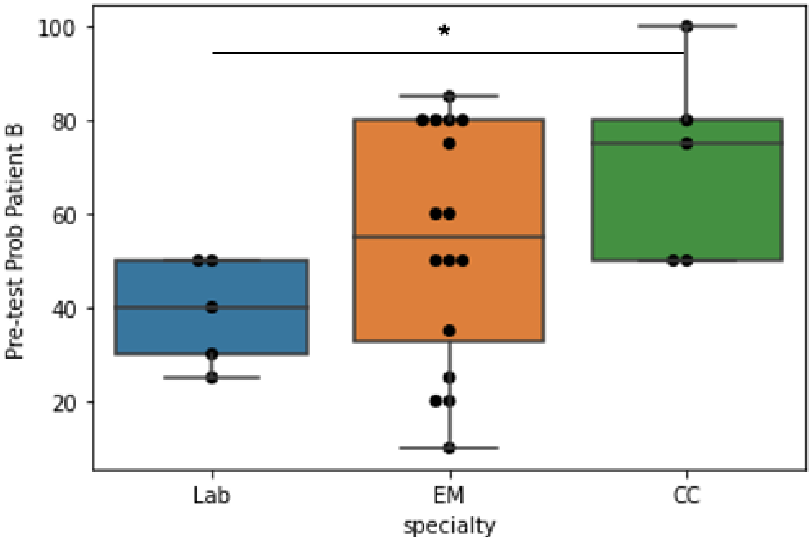

Supplemental Table S1. Response to IntelliSep “Band 1” Delphi Statements

Delphi Statements	Response for Patient A	Response for Patient B
If an IntelliSep test results in a “Band 1” score, I would consider:*		
- A lower level of care (e.g., Observation status, Discharge from the ED with 48h follow-up after alternative diagnoses that would require admission have been ruled out)	-	N/A
- A lower level of care (e.g., Admission to the Floor - if appropriate or Observation status)	N/A	-
- Continuing sepsis-related workup (watchful waiting)	-	-
- A high likelihood of an alternate diagnosis (other than sepsis)	✓	-
- Initiating infectious source identification and control	-	-
- Initiating a short and/or narrow course of antibiotics	-	-
- Withholding any form of antibiotics	-	N/A
- Starting fluid resuscitation as per internal sepsis protocol	-	-
- Ordering blood cultures	-	-
- Put additional effort into finding an alternative diagnosis (vs. sepsis)	✓	-
- Weigh the risks of anti-microbial stewardship in the determination of antibiotic protocol (e.g., Could influence my decision to select a shorter duration, narrow spectrum, oral vs. IV)	-	✓
- Reconsider aggressive fluid resuscitation, given heart failure history	N/A	✓
- Reassess mental status change after fluid bolus	✓	N/A
- Reconsider the need for additional sepsis-driven testing	✓	-
- Reconsider the need for a sepsis-related consult	N/A	-
- PCT testing	-	-
If I already had a negative PCT test result (negative defined as levels <0.50 ng/mL), that coupled with an IntelliSep Band 1 score for the patient would give me the added confidence to consider a more conservative approach as it relates to sepsis-focused care.	-	-

Symbols: ✓ Agree, X Disagree, - No consensus, N/A Not asked
** For Patient A, consensus was reached for 1 of 10 Band 1 statements in questionnaire #1, and 3 of 4 Band 1 statements in questionnaire #2. For Patient B, consensus was reached for 0 of 9 Band 1 statements in questionnaire #1, and 2 of 5 Band 1 statements in questionnaire #2.*

Supplemental Table S2. Response to IntelliSep “Band 3” Delphi Statements

Delphi Statements	Response for Patient A	Response for Patient B
If an IntelliSep test results in a “Band 3” score, I would consider:*		
- A higher level of care (e.g. admission to the step-down unit, ICU, etc.)	-	✓
- Immediate aggressive sepsis-focused care	✓	✓
- Continuing sepsis-related workup (watchful waiting)	-	-
- A high likelihood of an alternate diagnosis (other than sepsis)	✗	-
- Initiating consultation(s) (e.g., Imaging, surgical consult, critical care consult, etc.)	-	-
- Initiating infectious source identification and control	✓	✓
- Immediate initiation of antibiotics	✓	✓
- Starting fluid resuscitation as per internal sepsis protocol	✓	-
- Ordering blood cultures	✓	✓
- Aggressive pathogen ID testing (e.g., Biofire syndromic panels, T2	-	-

panels, etc)		
- PCT testing	-	-
If I already had a negative PCT test result (negative defined as levels <0.50 ng/mL), that coupled with an IntelliSep Band 3 score for the patient would give me the added confidence to consider a more conservative approach as it relates to sepsis-focused care.	-	-

Symbols: ✓ Agree, X Disagree, - No consensus, N/A Not asked

Supplemental Table S3. Response to IntelliSep “Band 2” Delphi Statements

Delphi Statements	Response for Patient A	Response for Patient B
If an IntelliSep test results in a “Band 2” score, I would consider:*		
- A higher level of care (e.g., admission to the step-down unit, ICU, etc.)	-	-
- Reconsider discharging this patient	-	-
- Immediate aggressive sepsis-focused care	-	-
- Continuing sepsis-related workup (watchful waiting)	-	-
- A high likelihood of an alternate diagnosis (other than sepsis)	-	-
- Initiating infectious source identification and control	✓	-
- Immediate initiation of antibiotics	✓	✓
- Starting fluid resuscitation as per internal sepsis protocol	-	-
- Ordering blood cultures	✓	-
- Aggressive pathogen ID testing (e.g., Biofire syndromic panels, T2 panels, etc.)	-	-
- Tracking the trajectory of the IntelliSep scores over time	-	-
- PCT testing	-	-
If I already had a negative PCT test result (negative defined as levels <0.50 ng/mL), that coupled with an IntelliSep Band 3 score for the patient would give me the added confidence to consider a more conservative approach as it relates to sepsis-focused care.	-	-

Symbols: ✓ Agree, X Disagree, - No consensus, N/A Not asked

Supplemental Table S4. Response to IntelliSep Delphi Statements for Patient C

Delphi Statements	Response for Patient C
If an IntelliSep test results in a Band 1 score for Patient C, I would consider:	
- Give additional consideration to an alternate diagnosis (other than sepsis)	✓
- Weigh the risks of anti-microbial stewardship in the determination of antibiotic protocol (e.g., Could influence my decision to select a shorter duration, narrow spectrum, oral vs. IV)	✓
- Consider more conservative fluid resuscitation	✓
- Reconsider the need for more exhaustive evaluation of source (advanced imaging, CT scan, etc.)	-
- Reconsider the need for a sepsis-related consult	-
If an IntelliSep test results in a Band 3 score for Patient C, I would consider:	
- Immediate aggressive sepsis-focused care	✓
- A high likelihood of an alternate diagnosis (other than sepsis)	✗
- Immediate initiation of antibiotics	✓
- Ordering blood cultures	✓
If an IntelliSep test results in a Band 2 score for Patient C, I would consider:	
- Immediate aggressive sepsis-focused care	-
- Continuing sepsis-related workup (watchful waiting)	✓
- A high likelihood of an alternate diagnosis (other than sepsis)	-
- Ordering blood cultures	-
- Tracking the trajectory of the IntelliSep scores over time (if data was available supporting the utility of doing so)	✓

Symbols: ✓ Agree, X Disagree, - No consensus, N/A Not asked

Details of the Delphi Study Design

Details regarding the design of the Delphi study that was discussed in this paper have been previously published. Publication citation and link to the paper is captured below, for reference.

Publication Citation: Kraus DO, C. K. et al. Rapid identification of sepsis in the emergency department. J Am Coll Emerg Physicians Open 4, e12984 (2023).

Publication Link: https://www.ncbi.nlm.nih.gov/pmc/articles/PMC10239543/
